# Tuft Cell Formation Reflects Epithelial Plasticity in Pancreatic Injury: Implications for Modeling Human Pancreatitis

**DOI:** 10.3389/fphys.2020.00088

**Published:** 2020-02-14

**Authors:** Kathleen E. DelGiorno, Razia F. Naeem, Linjing Fang, Chi-Yeh Chung, Cynthia Ramos, Natalie Luhtala, Carolyn O’Connor, Tony Hunter, Uri Manor, Geoffrey M. Wahl

**Affiliations:** ^1^Gene Expression Laboratory, Salk Institute for Biological Studies, La Jolla, CA, United States; ^2^Waitt Advanced Biophotonics Center, Salk Institute for Biological Studies, La Jolla, CA, United States; ^3^Molecular and Cell Biology Laboratory, Salk Institute for Biological Studies, La Jolla, CA, United States; ^4^Flow Cytometry Core, Salk Institute for Biological Studies, La Jolla, CA, United States

**Keywords:** tuft cells, Dclk1, pancreatitis, metaplasia, mouse models

## Abstract

Chronic pancreatitis, a known risk factor for the development of pancreatic ductal adenocarcinoma (PDA), is a serious, widespread medical condition characterized by inflammation, fibrosis, and acinar to ductal metaplasia (ADM). ADM is a cell type transdifferentiation event where pancreatic acinar cells become ductal-like under conditions of injury or oncogenic mutation. Here, we show that chronic pancreatitis and ADM in genetically wild type mice results in the formation of a significant population of chemosensory tuft cells. Transcriptomic analyses of pancreatitis tuft cells identify expression of inflammatory mediators, consistent with a role for tuft cells in injury progression and/or resolution. Though similar to tuft cell populations in other organs and disease systems, we identified a number of key differences that suggest context-specific tuft cell functions. We evaluated seven different mouse strains for tuft cell formation in response to chronic injury and identified significant heterogeneity reflecting varying proclivity for epithelial plasticity between strains. These results have interesting implications in the role of epithelial plasticity and heterogeneity in pancreatitis and highlight the importance of mouse strain selection when modeling human disease.

## Introduction

Pancreatitis, the third leading cause of gastrointestinal-related hospitalizations, is a serious medical condition characterized by inflammation of the exocrine pancreas. Major risk factors include gallstones and alcohol abuse; however, pancreatitis can also result from use of certain medications, autoimmune diseases, infection, trauma, metabolic disorders, surgery, or mutations in genes associated with digestive enzymes (such as *Prss1*) (Whitcomb, 2013). Pancreatitis presents in either an acute or chronic form. Acute pancreatitis is a painful, short-term condition with sudden onset that typically resolves within a few days following treatment, but can be fatal in severe cases associated with multi-organ failure (Murtaugh and Keefe, 2015; Yang and Forsmark, 2017). By contrast, chronic pancreatitis is largely asymptomatic, occurs gradually, and is typically diagnosed only after the presentation of complications. In its chronic form, pancreatitis is characterized by persistent inflammation, fibrosis, and acinar cell metaplasia and results in permanent pancreatic damage (Murtaugh and Keefe, 2015). Repeated episodes of acute pancreatitis can progress to chronic pancreatitis. While both forms carry a risk of mortality, they can also lead to other serious conditions. For example, chronic pancreatitis is a known risk factor for pancreatic ductal adenocarcinoma (PDA), currently the third-most common cause of cancer-related death in the United States (Pinho et al., 2014; Siegel et al., 2019).

The exocrine compartment of a healthy pancreas is comprised largely of acinar cells, which produce and secrete digestive enzymes and ductal cells, which transport these enzymes to the duodenum. Under conditions of chronic injury or oncogenic mutation, differentiated acinar cells undergo metaplasia, or a morphological remodeling, to form ductal-like cells expressing developmental factors, thought to represent a facultative progenitor population (Storz, 2017). This acinar to ductal metaplasia (ADM) is a reversible process thought to play a role in tissue healing and regeneration after injury (Storz, 2017). In the presence of persistent oncogenic *Kras^*G*12*D*^* expression, however, metaplastic cells are no longer able to re-differentiate to acinar cells, and instead progress to pancreatic intraepithelial neoplasias (PanINs) and PDA (Storz, 2017).

We previously showed that *Kras^*G*12*D*^*-induced ADM is characterized by substantial cellular heterogeneity, including a significant population of tuft cells (Delgiorno et al., 2014). Tuft cells are solitary chemosensory cells found in the hollow organs of the respiratory and digestive tracts and can be identified by their large microvilli and deep actin rootlets in the supranuclear cytoplasm (Sato, 2007). Tuft cell formation has been identified in response to tissue injury and tumorigenesis in a number of organs including the colon, lung, and stomach (Filippenko, 1981; Gerbe et al., 2011; Saqui-Salces et al., 2011). Tuft cells are associated with human pancreatitis, but have not previously been identified in genetically wild-type mouse models of pancreatic injury (Delgiorno et al., 2014).

Despite the discovery of this striking cell type in 1956, little functional data existed until recently (Jarvi and Keyrilainen, 1956; Rhodin and Dalhamn, 1956). Several groups have independently shown that intestinal tuft cells sense and repel parasite infection through the expression of succinate receptor 1 and secretion of cytokine IL-25 (Gerbe et al., 2011; Howitt et al., 2016; von Moltke et al., 2016; Lei et al., 2018; Nadjsombati et al., 2018; Schneider et al., 2018). Further, thymic tuft cells have been shown to play a role in the development and polarization of thymic invariant natural killer T cells (Bornstein et al., 2018; Miller et al., 2018). While these data demonstrate a role for tuft cells in mediating inflammation, their role in tissue injury represents a substantial knowledge gap.

In contrast to the intestines and thymus, the murine pancreas is normally devoid of tuft cells. Rather, pancreatic tuft cells emerge in response to expression of tumor-initiating mutations (Bailey et al., 2014; Delgiorno et al., 2014). Despite this unexpected discovery, we previously lacked the genetic tools and mouse models to properly study tuft cell function in the pancreas. This led tuft cell function to be predicted largely on the basis of transcriptomic and protein expression studies done in the intestines. For example, in intestines, high expression of the tubulin kinase Dclk1 is largely restricted to tuft cells (Gerbe et al., 2011). In the pancreas, however, Westphalen et al. (2016) have shown by lineage tracing with BAC transgenic Dclk1-CreERT mice that Dclk1 labeling is not tuft cell-restricted as it also labels acinar and ductal cells. This is consistent with acinar cell plasticity and the reliance of the pancreas on metaplasia to heal. Intermediate cell types that exhibit acinar and ductal characteristics are generated during metaplasia and preclude the use of a single marker for tuft cell identification.

Here, we demonstrate that tuft cell formation occurs during ADM in response to chronic injury in multiple strains of genetically wild type mice. We characterize this pancreatitis tuft cell population using a combination of histology, fluorescence and electron microscopy, and molecular strategies. Transcriptomic characterization of pancreatitis tuft cells reveals both similarities and differences to those found in other organs and disease systems. Our data demonstrate that tuft cell formation is a normal part of pancreatic injury and suggest a role in recovery. Interestingly, we found tuft cell formation and loss of acinar identity to be strain-specific. We have found that a proportion of human pancreatitis cases are characterized by tuft cell formation (Delgiorno et al., 2014). Therefore, these data highlight the importance of using the appropriate mouse strain and conditions to more accurately model the human condition.

## Materials and Methods

### Animal Strains

Mice were housed in accordance with NIH guidelines in Association for Assessment and Accreditation of Laboratory Animal Care (AAALAC)-accredited facilities at the Salk Institute for Biological Studies. The Institutional Animal Care and Use Committee at the Salk Institute approved all animal studies. C57BL/6J mice were either purchased from The Jackson Laboratory or bred in-house. Eight-weeks-old DBA/2, Swiss Webster, BALB/c, and FVB mice were purchased from Charles River Laboratories. CD-1 mice were either purchased from Charles River Laboratories or bred in-house. Eight-weeks-old 129S6 mice were purchased from Taconic Biosciences. *Ptf1aCre*^*ERTM/+*^ and *Rosa*^*YFP*^ strains have been previously described and were purchased from The Jackson Laboratory (Pan et al., 2013). FLARE25 (*Il25^*F*25/F25^*) mice (C57Bl/6J strain) were generously provided by the Locksley (University of California at San Francisco, CA, United States) and von Moltke (University of Washington, WA, United States) laboratories and were bred to the CD-1 mouse strain (von Moltke et al., 2016). F1 mice were used for experiments.

### Pancreatitis Induction

Pancreatitis was induced by intraperitoneal (IP) injection of caerulein (Bachem). One cycle includes twice daily treatment, 5 days a week at 125 μg/kg with 2 days recovery. Mice in Supplementary Figure S3 were treated with 62.5 μg/kg, 125 μg/kg, or 250 μg/kg caerulein.

### Lineage Tracing

Lineage tracing was conducted using the *Ptf1aCre*^*ERTM/+*^; *Rosa*^*YFP*^ mouse model, as previously described (Pan et al., 2013; Delgiorno et al., 2014). Mice were bred into the CD-1 mouse strain; F4 mice were used. In this model, tamoxifen treatment induces Cre activity, which then initiates expression of yellow fluorescent protein (YFP) specifically in *Ptf1a* + acinar cells. Acinar cells were labeled in *Ptf1aCre*^*ERTM/+*^; *Rosa*^*YFP*^ mice with five daily doses of 5 mg tamoxifen (Sigma, 5 days/week for 2 weeks) delivered in corn oil (Sigma) by oral gavage. Pancreatitis was then induced with four cycles of 250 μg/kg caerulein.

### Histological Staining and Quantification

Tissues were fixed overnight in zinc-containing neutral-buffered formalin (Fisher Scientific), embedded in paraffin, cut in 5 μm sections, mounted, and stained. Sections were deparaffinized in xylene, rehydrated in a series of ethanol, and then washed in PBST and PBS. Endogenous peroxidase activity was blocked with a 1:50 solution of 30% H_2_O_2_: PBS followed by microwave antigen retrieval in 100 mM sodium citrate, pH 6.0. Sections were blocked with 1% bovine serum albumin (BSA) and 5% goat or rabbit serum in 10 mM Tris (pH 7.4), 100 mM MgCl_2_, and 0.5% Tween-20 for 1 h at room temperature, followed by an avidin/biotin blocking kit (Thermo Fisher Scientific) per the manufacturer’s instructions. Primary antibodies were diluted in blocking solution and incubated overnight. Information on primary antibodies is provided in Supplementary Table S1. Slides were then washed, incubated in streptavidin-conjugated secondary antibodies (for rabbit or mouse antibodies, Abcam, for rat or goat antibodies, Vector) and developed with DAB substrate (Vector). Hematoxylin and eosin (H&E) staining was done to assess tissue morphology. All slides were scanned and imaged on an Olympus VS-120 Virtual Slide Scanning microscope. For quantification of histology, ten 20× fields per scanned slide were scored in a blinded fashion using the ImageJ/FIJI plugin immunohistochemistry (IHC) image analysis toolbox (Shu et al., 2013). A statistical color detection model was trained based on multiple regions of interest (ROIs) manually and selected from desired color pixel regions from sample images for each strain using the IHC Toolbox plugin. Each image was color deconvolved using its corresponding trained model within the plugin and a new RGB image containing only the isolated color was automatically generated. The hematoxylin counter stain was deconvolved in a similar manner. Using ImageJ/FIJI, the desired color-isolated image and the counter stain-isolated image was binarized and staining area of the two was measured by counting the number of pixels of foreground (Schindelin et al., 2012). The percentage of signal was determined by dividing the stain area by the sum of the stain area and the counter stain.

### Fluorescence Microscopy

Immunofluorescence on paraffin-embedded tissues followed the IHC protocol until the blocking step. Instead, tissues were blocked with 5% normal donkey serum and 1% BSA in 10 mM PBS for 1 h at room temperature. Tissue sections were stained with primary antibodies in 10 mM PBS supplemented with 1% BSA and 0.1% Triton X-100 overnight (Supplementary Table S1). Sections were then washed 3 × 15 min in PBS with 1% Triton X-100, incubated in Alexa Fluor secondary antibodies and/or phalloidin (Invitrogen), washed again for 3 × 5 min, rinsed with distilled water, and mounted with Prolong Gold containing Dapi (Invitrogen). Immunofluorescence on OCT-embedded sections was conducted as previously described (Delgiorno et al., 2014). Tissues were imaged on a Zeiss 710 confocal microscope, a Zeiss 880 Airyscan Super-Resolution microscope or an Olympus VS-120 Virtual Slide Scanning microscope.

### Multiplex Immunofluorescence

Co-expression of amylase and pan-cytokeratin (antibodies, Supplementary Table S1) was determined using a Perkin Elmer Opal 4-color Manual IHC Kit (NEL810001KT) per the manufacturer’s instructions.

### Transmission Electron Microscopy

Tissues were fixed in a solution of 2% paraformaldehyde, 2.5% glutaraldehyde, and 2 mM CaCl_2_ in 0.15 M sodium cacodylate buffer (pH 7.4) for 2 h at room temperature. They were then post-fixed in 1% osmium tetroxide for 40 min and 1.5% potassium ferricyanide in sodium cacodylate buffer for 1 h at 4°C in the dark. Tissues were stained en block in 1% aqueous uranyl acetate (4°C in the dark), dehydrated in a series of graded ethanol, and embedded in Eponate12 resin (Ted Pella). Ultra-thin sections (70 nm) were obtained using a diamond knife (Diatome) in an ultramicrotome (Leica EM UC7) and placed on copper grids (300 mesh). Sections were imaged on a Zeiss Libra 120 TEM operated at 120 kV.

All images (IHC, IF, and EM) were digitally enhanced to edit the color, brightness, and contrast levels using Zen (Carl Zeiss) and/or Photoshop (Adobe) software.

### Tuft Cell Preparation and Cell Sorting

Pancreatitis tuft cells were isolated from wild-type CD-1 mice treated with four cycles of 250 μg/kg caerulein (allowed to recover for 0–2 days). The pancreas was quickly dissected, minced in 5 ml of DMEM with FBS and allowed to incubate for 2 min. Supernatant and fat were removed and pancreatic tissue was then incubated in 10 ml DMEM supplemented with 1 mg/ml collagenase I (Sigma), 1 mg/ml soybean trypsin inhibitor (Gibco), 1 mg/ml hyaluronidase (Sigma), and 250 μl of DNAse I, shaking gently at 37°C for a maximum of 30 min. Digestion was monitored and tissue was further digested mechanically by pipetting. Digested tissue was passed through a 100 μm filter, washed with fluorescence activated cell sorting (FACS) buffer (PBS, 1 mM EDTA, 0.5% BSA), and incubated with ACK lysing buffer (Gibco) to remove red blood cells before staining for FACS.

Single cell suspensions were incubated on ice with mouse Fc receptor block (BD Biosciences, 1:200) followed by antigen-specific antibodies in FACS buffer. DAPI (molecular probes, 1:1000) and Annexin V (Biolegend, Pacific Blue conjugate at 1:200) were used to exclude dead and dying cells. Cells were labeled with Cd45 (Alexa Fluor 488), EpCAM (Alexa Fluor 647), and Siglec F (PE) (Biolegend, 1:200) antibodies. Fluorescence minus one (FMO) staining controls were included for gating populations of interest. Cells were FACS purified at the Salk Institute’s Flow Cytometry core facility on a BD Biosciences Influx cell sorter (100-μm size nozzle, 1 × PBS sheath buffer with sheath pressure set to 20 PSI). Cells were sorted in 1-drop Single Cell sort mode for counting accuracy; these were deposited directly into lysis buffer composed of DNase/RNase-free water, (%) Triton X-100, and Ribolock (Thermo Fisher Scientific) in a 96 well plate.

### RNA-seq Library Generation, High-Throughput Sequencing, and Analysis

Low input bulk RNA sequencing (RNA-seq) on pancreatitis tuft cells was performed using the Smart-Seq2 protocol as previously described (Picelli et al., 2014). In brief, five biological replicates, each with 100 tuft cells from an individual mouse, were sorted directly into 2 μl of Smart-Seq2 lysis buffer. Full-length cDNA was generated and size distribution was checked with Agilent TapeStation 4200 to ensure RNA quality. cDNA were then amplified with 18–22 PCR cycles, tagmentated, and amplified again with 10 PCR cycles using a Nextera XT kit (Illumina FC-131-1096). The sequencing library was purified with AMPure XP beads. 50 bp single-end sequencing was performed with Illumina HiSeq 2500. Sequencing reads were quality checked with FastQC and mapped to the mouse genome (mm9) using Hisat2 (Kim et al., 2015). Transcript assembly and quantification were performed by Stringtie and Ballgown (Pertea et al., 2016). Gene expression distribution between samples was checked to ensure similar transcriptome quality. Genes that have RPKM variance across samples <1 were first removed to exclude non-expressed genes. The RPKM values were then Log2 transformed and quantile normalized with the R package preprocessCore. Differential expression analysis between tuft and non-tuft epithelial cells was performed with empirical Bayes shrinkage and moderated *t*-test using the Limma package and *p*-values were converted into false discovery rates (FDR) using the Benjamini-Hochberg procedure (Ritchie et al., 2015). Heat map plotting and clustering (with Euclidean distance and complete linkage) was performed with heatmap.2 in R. The differential expression score was calculated as: −Log_10_ (FDR) × Log_2_ (fold change), which factors in both statistical confidence and the effect size. Gene ontology network analyses of differentially expressed genes were performed with ClueGO plug-in of Cytoscape (Bindea et al., 2009).

### RT-qPCR

RT-qPCR was performed using Power SYBR Green PCR Master Mix (Applied Biosystems) on the ABI 7900 detection system (Applied Biosystems). Relative expression values were determined using the standard curve method. RT-qPCR was performed on bulk tuft cells and non-tuft epithelial cells from CD-1 mice with pancreatitis (100 cells per group) after the amplification step of the SmartSeq2 protocol (Picelli et al., 2014). Results were normalized to the housekeeping gene Rplp0. Primer sequences can be found in Supplementary Table S2.

## Results

### Chronic Pancreatic Injury Induces Tuft Cell Formation

Tuft cell formation is characteristic of a portion of human pancreatitis cases and has also been shown in multiple mouse models of tissue injury in several gastrointestinal organs (Saqui-Salces et al., 2011; Delgiorno et al., 2014). We and others have shown that tuft cells form in the pancreas in response to genetic modifications, such as oncogenic *Kras^*G*12*D*^* or TGFα, Sox17, or IL-1β overexpression (Bailey et al., 2014; Delgiorno et al., 2014; Westphalen et al., 2016). Importantly, studies of the generality of tuft cell formation in genetically wild type mice have yet to be reported. Therefore, we conducted a multiparameter analysis of caerulein-induced mouse pancreatitis.

**FIGURE 1:**
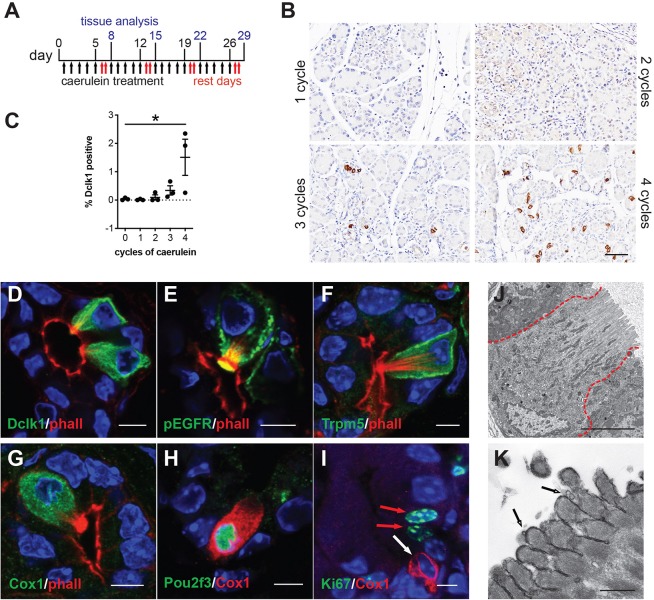
Chronic injury induces tuft cell formation in the pancreata of wild type mice. **(A)** Schematic of caerulein treatment for pancreatitis induction in wild type mice. **(B)** Immunohistochemistry (IHC) demonstrating an increase in Dclk1 expression (brown) in the injured pancreas over time and quantified in **(C)** (*n* = 3 mice per condition). Scale bar, 50 μm. **(D)** Co-immunofluorescence (Co-IF) for tuft cell marker phalloidin (red, labels the microvilli and actin rootlets of tuft cells) and Dclk1, **(E)** phospho-EGFR, **(F)** Trpm5, or **(G)** Cox1 (green). **(H)** Co-IF for tuft cell marker Cox1 (red) and Pou2f3 or **(I)** Ki67 (green). Red arrows, Ki67 + nuclei, white arrow, Ki67- tuft cell. Scale bars, 5 μm. **(J)** Electron microscopy confirming tuft cell formation in pancreatitis, and **(K)** demonstrating the presence of vesicles at the tips of the microvilli (arrows). Scale bars, 5 μm and 500 nm, respectively.

Caerulein is a cholecystokinin ortholog that induces endoplasmic reticulum (ER) stress in pancreatic acinar cells, resulting in cell death, inflammation, and edema (Sah et al., 2014). Previously, we evaluated tuft cell formation in response to caerulein using mixed background mice and 2 weeks (two cycles) of treatment and were unable to detect a robust response (Delgiorno et al., 2014). To more thoroughly evaluate the potential for tuft cell genesis, we varied treatment dose and timing in outbred CD-1 mice. We found that prolonged caerulein treatment resulted in acinar cell loss and an increase in ADM, stromal deposition, and mucin expression (Supplementary Figure S1). By IHC, we identified robust up-regulation of Dclk1 + cells with tuft cell morphology after four cycles of caerulein treatment (Figures 1A–C). Interestingly, this response was enhanced with increasing caerulein dosage and length of time of treatment (data not shown). We confirmed tuft cell identity using co-immunofluorescence for additional tuft cell markers and F-actin marker phalloidin, which labels the deep actin rootlets and striking microvilli characteristic of this cell type (Figures 1D–G; Delgiorno et al., 2014). This analysis revealed that cells with high Dclk1 expression are tuft cells (100%+, 130/130 cells, 3 mice) (Figure 1D). We further confirmed tuft cell genesis with markers phospho-EGFR, a receptor tyrosine kinase (100%+, 128/128 cells, 3 mice), transient receptor potential cation channel, subfamily M, member 5 (Trpm5) a chemosensory cell marker (100%+, 110/110 cells, 3 mice), and Cox1, a prostaglandin synthase (95%+, 218/230, 3 mice) (Figures 1E–G; Bezencon et al., 2008; Gerbe et al., 2011; Delgiorno et al., 2014). Pancreatitis-induced tuft cells (Cox1+) express tuft cell master regulator Pou2f3 (97%+, 189/195 cells, 4 mice), and consistent with *Kras^*G*12*D*^*-induced tuft cells, are not proliferative, (0% Ki67+, 150/150 cells, 3 mice) (Figures 1H,I; Delgiorno et al., 2014; Gerbe et al., 2016). Taken together, these data reveal tuft cell genesis in response to chronic, long-term injury in the outbred CD-1 mouse strain.

Next, we used transmission electron microscopy (TEM) to explore the ultrastructure of pancreatitis-induced tuft cells. We induced pancreatic injury and tuft cell formation with four cycles of caerulein. We then confirmed tuft cell formation by the presence of cells with characteristic microvilli, actin rootlets, and abundant intracellular vesicles and mitochondria (Figure 1J). Closer examination revealed membrane-bound electron lucent vesicles, which appear to be budding from the tips of the microvilli, consistent with injury-induced tuft cells being a secretory cell type (Figure 1K). Collectively, these data identify *de novo*, spontaneous tuft cell formation in chemically induced pancreatic injury. The data further suggest a potential secretory role for pancreatic tuft cells in inflammation. To further evaluate this role, we conducted RNA sequencing (RNA-seq).

### Transcriptomic Analysis of Pancreatitis Tuft Cells

**FIGURE 2:**
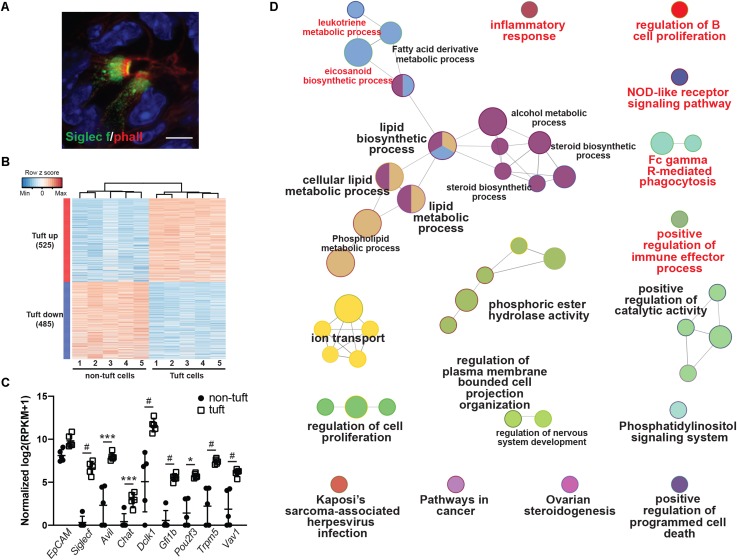
Transcriptomic analysis of pancreatitis-induced tuft cells in wild type CD1 mice. **(A)** Co-immunofluorescence for phalloidin (red, labels the microvilli and actin rootlets of tuft cells) and Siglec f (green) in the injured pancreas. Scale bar, 5 μm. **(B)** Heat map with hierarchical clustering showing differentially expressed genes in EpCAM+; Siglec f+ tuft cells as compared to EpCAM+; Siglec f-neg non-tuft epithelial cells. **(C)** Confirmation of Siglec f expression and tuft cell markers in sorted EpCAM+; Siglec f+ cells. *n* = 5 mice. **(D)** Gene ontology network analysis of genes differentially up-regulated in tuft cells, as compared to non-tuft epithelial cells, identifies immune cell signaling pathways (labeled in red). *p < 0.05; ***p < 0.005; #*p* < 0.001.

To provide a comprehensive, agnostic, molecular assessment of the potential role(s) for tuft cells in pancreatic injury, we used RNA-seq and bioinformatic analyses. To accomplish this, we induced pancreatitis with caerulein, collected tuft cells by FACS, and then conducted RNA-seq. Siglec f is a cell surface lectin that is typically used to detect eosinophils, but expression is also evident in intestinal tuft cells (Gerbe et al., 2016). Using co-immunofluorescence with phalloidin, we found that Siglec f also labels pancreatitis-induced tuft cells in wild type CD-1 mice (98.6%+, 276/280 cells, *n* = 3 mice) (Figure 2A).

As tuft cells are rare in pancreatitis, we employed a small cell number sequencing approach. We used FACS to isolate 100 Cd45-neg; EpCAM+; Siglec f+ tuft cells and an equivalent number of Cd45-neg; EpCAM+; Siglec f-neg non-tuft epithelial cells from five mice with pancreatitis (Supplementary Figure S2A). Tuft cells constituted approximately 0.80% of the epithelium (range 0.2–1.7%). We then isolated bulk RNA, prepared cDNA, and amplified it using SmartSeq2 methodology (Picelli et al., 2014). RNA-seq identified 1010 genes as differentially expressed between tuft cells and non-tuft epithelial cells in pancreatitis (Figure 2B; FDR < 0.05 and average fold change >4). Tuft cell isolation and enrichment was confirmed in the Siglec f+ population by high expression of tuft cell markers such as *Dclk1*, *Pou2f3*, and *Trpm5* (Figure 2C), and low-to-no expression of acinar cell markers, such as amylase genes, or islet cell markers, such as chromogranin A and somatostatin (Supplementary Figures S2B,C). Similar to tuft cells characterized in other organs, pancreatitis-associated tuft cells express a number of genes associated with bacterial or viral infection (Supplementary Figure S3). In contrast to intestinal tuft cells, *Sucnr1* was not expressed (Nadjsombati et al., 2018). Interestingly, network analysis of genes differentially up-regulated in tuft cells identified a number of immune cell signaling pathways, consistent with a role in inflammation (Figure 2D and Supplementary Figure S3). These data are consistent with a sentinel cell role for pancreatitis tuft cells in innate immunity.

### Pancreatitis Tuft Cells Express Cytokine IL-25

**FIGURE 3:**
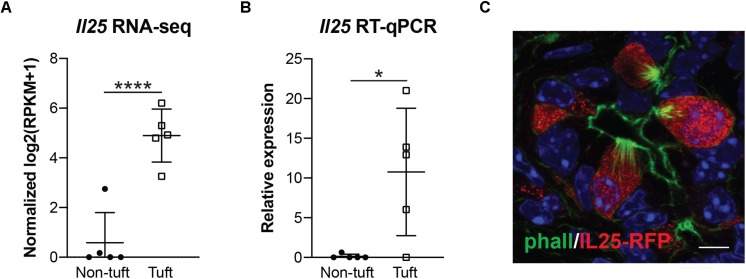
Pancreatitis-induced tuft cells in wild type CD1 mice express cytokine IL-25. **(A)** RNA-seq demonstrating significantly higher expression of *Il25* in tuft cells (EpCAM+; Siglec f+ cells) as compared to non-tuft epithelial cells (EpCAM+; Siglec f-neg cells). **(B)** RT-qPCR confirms significantly higher *Il25* expression in tuft cells. **(C)** Co-immunofluorescence for red fluorescent protein (RFP, red) and phalloidin (green) in *Il25^*F*25/F25^* mice with pancreatitis. Scale bar, 5 μm. *p < 0.05; ****p < 0.001.

Interleukin 25 (IL-25, also called IL-17E) is a member of the IL-17 cytokine superfamily that has been demonstrated to both induce and enhance the helper T cell 2 (Th2) immune response while inhibiting Th1 and Th17-driven immunity. Expression has been shown in many different cell types, tissues, and systems, among which are tuft cells (Bezencon et al., 2008; von Moltke et al., 2016). Within the small intestine, tuft cell-derived IL-25 is required for the “weep and sweep” response that rids the host of helminth infection (Gerbe et al., 2016; Howitt et al., 2016; von Moltke et al., 2016). Given the established role for this cytokine as a tuft cell effector, we used several orthogonal methods to determine if IL-25 is expressed in pancreatitis tuft cells. By RNA-seq, we found *Il25* expression to be significantly higher in tuft cells than non-tuft epithelial cells (Figure 3A), which was confirmed by RT-qPCR (Figure 3B). To further confirm IL-25 expression, we induced chronic injury in IL-25 reporter mice (Flare25, flox and reporter of *Il25; Il25^*F*25/F25^*). By co-immunofluorescence with phalloidin, we found that 100% of tuft cells analyzed (101/101 cells, *n* = 5 mice) were positive for red fluorescent protein (RFP) and IL-25 expression (Figure 3C). These data suggest a potential role for tuft cell-derived IL-25 in pancreatitis.

### Pancreatitis Tuft Cells Result From Acinar to Ductal Metaplasia

**FIGURE 4:**
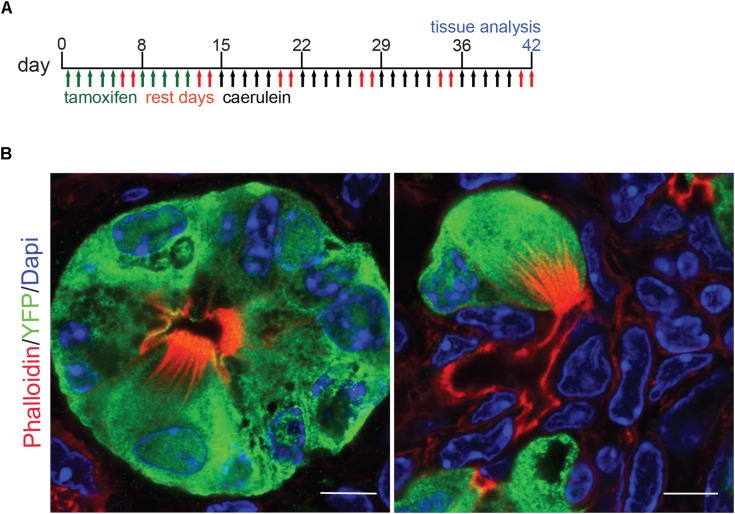
Lineage tracing of pancreatitis tuft cells in wild type mice reveals acinar cell origin. **(A)** Schematic of tamoxifen and caerulein treatment for lineage tracing in *Ptf1aCre*^*ERTM/+*^; *Rosa*^*YFP*^ mice. **(B)** Co-immunofluorescence for tuft cell marker phalloidin (red, labels the microvilli and actin rootlets of tuft cells) and YFP, green, in caerulein-treated pancreata. Scale bar, 5 μm.

Previously, we used lineage tracing to show that the majority of *Kras^*G*12*D*^*-induced pancreatic tuft cells result from acinar-to-ductal metaplasia (ADM, 68.7%) (Delgiorno et al., 2014). To determine if pancreatitis tuft cells transdifferentiate from the acinar cell epithelium in response to injury, we conducted lineage tracing using *Ptf1aCre^*ERTM/+*^;Rosa^*YFP*^* mice crossed into the CD-1 background. In this model, tamoxifen treatment induces Cre activity and expression of YFP, specifically in *Ptf1a*+ adult acinar cells (Pan et al., 2013). Adult mice were given tamoxifen followed by four cycles of caerulein treatment to induce pancreatitis and tuft cell formation (Figure 4A). To identify tuft cells, we conducted co-immunofluorescence with phalloidin and observed YFP expression in 97.6% of tuft cells analyzed (203/208 cells, *n* = 2 mice), signifying that injury-induced pancreatitis tuft cells can arise from acinar cells (Figure 4B). The other 2.4% of tuft cells may not have expressed YFP due to incomplete recombination, reporter methylation, or additional cell(s) of origin (ductal cells, etc.). Interestingly, although significantly lower than in non-tuft epithelial cells, a number of acinar cell genes were detected in our Siglec f+ tuft cell population by RNA-seq (Supplementary Figure S2B), perhaps reflecting their acinar cell of origin.

### Tuft Cell Formation Is Mouse Strain-Dependent

**FIGURE 5:**
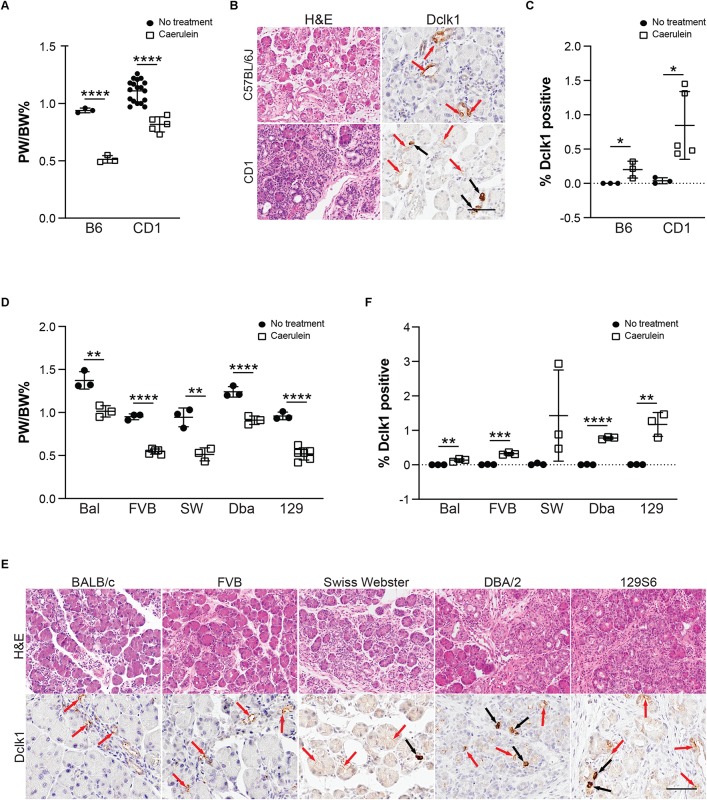
Tuft cell formation in response to pancreatitis in wild type mice is strain-dependent. **(A)** Pancreas: Body weight ratio (PW/BW) changes in C57BL/6J (B6) and CD1 mice in response to four cycles of caerulein treatment. **(B)** H&E and IHC for tuft cell marker Dclk1 in caerulein-treated C57BL/6J and CD1 mice showing expression consistent with tuft cell morphology (black arrows) as well as non-tuft cells (red arrows), quantified in **(C)**. **(D)** Pancreas: Body weight ratio (PW/BW) changes in an additional five mouse strains in response to four cycles of caerulein treatment. **(E)** H&E and IHC for tuft cell marker Dclk1 in the same caerulein-treated mouse strains. Cells with tuft cell morphology (black arrows), non-tuft cells (red arrows). Dclk1 IHC quantified in **(F)**. Bal, BALB/c; SW, Swiss Webster; Dba, DBA/2; 129, 129S6. Scale bar, 50 μm. *p < 0.05, **p < 0.01; ***p < 0.005; ****p < 0.001.

To determine the function of tuft cells in pancreatitis, we sought to employ a number of genetically engineered mouse models (GEMMs) that would allow us to eliminate tuft cell formation and/or secreted effectors. For the purpose of standardization, however, most GEMMs are typically backcrossed into the inbred C57Bl/6J mouse strain. To first confirm that C57Bl/6J can generate tuft cells in response to chronic injury, we induced pancreatitis with 1–5 cycles of caerulein treatment. Surprisingly, we found that, although C57Bl/6J mice develop pancreatitis and suffer significant loss of pancreas tissue in response to caerulein, similar to CD-1 (Figures 5A,B), we were unable to detect Dclk1+ cells with tall columnar, tuft cell morphology (Figures 5B,C). To determine if this strain exhibits reduced sensitivity to caerulein treatment or has a sex-specific response, we varied the dose of caerulein (four cycles of either 62.5 μg/kg or 250 μg/kg) and induced injury in both genders (Supplementary Figure S4). We found that increasing caerulein dosage leads to the formation of a few cells with tuft cell morphology, but to a lesser extent than identified in CD-1 mice (Supplementary Figure S4C). To determine if injury-induced tuft cell formation is unique to CD-1 mice, we evaluated pancreatitis in an additional five mouse strains. In total, we examined seven strains of mice: C57Bl/6J, BALB/c, FVB, CD-1, Swiss Webster, DBA/2, and 129S6. A minimum of three mice from each strain were treated with four cycles of caerulein and examined histologically. All treated mice experienced a decrease in pancreas weight to body weight ratio compared to control mice (of the same strain), and tissue injury and inflammation could be identified by H&E analysis in all strains (Figures 5D,E and Supplementary Figure S5). Interestingly, we found a significant increase in Dclk1 expression, identified by IHC, in nearly all strains (Figure 5F). However, expression levels varied and cellular morphology was often inconsistent with that of tuft cells, demonstrating that Dclk1 is not tuft cell specific (Figures 5E,F). Consistent with this, we identified Dclk1 staining in the normal ducts of the pancreas within all strains, which lack tuft cells. Importantly, and consistent with prior reports, these ducts lack tuft cells (Supplementary Figure S5; May et al., 2010; Delgiorno et al., 2014; Westphalen et al., 2016). Further, our histological and RNA-seq analyses are consistent in showing that while Dclk1 expression is significantly higher in tuft cells, it is not specific to them (Figure 2C).

**FIGURE 6:**
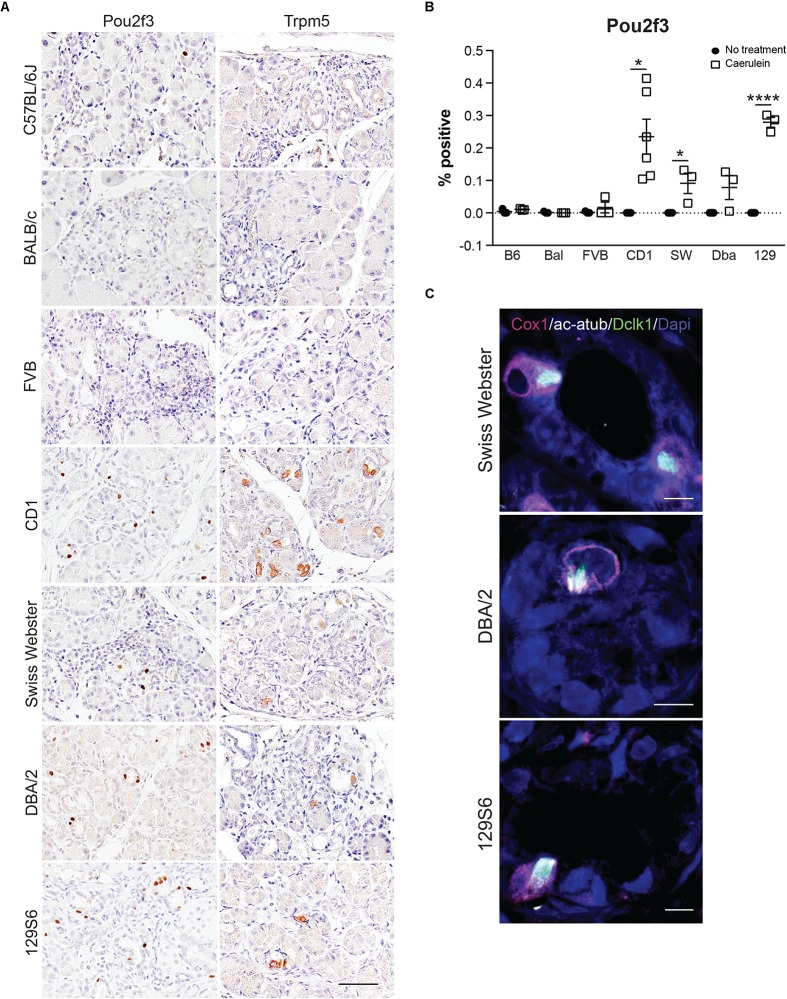
Tuft cell marker expression in response to pancreatitis in wild type mice is strain-dependent. **(A)** IHC for tuft cell markers Pou2f3 and Trpm5 in all strains. Scale bar, 50 μm. **(B)** Quantification of Pou2f3 IHC in all seven strains of mice. **(C)** Co-immunofluorescence for tuft cell markers Cox1 (pink), acetylated α-tubulin (white), and Dclk1 (green). Scale bar, 5 μm. Bal, BALB/c; SW, Swiss Webster; Dba, DBA/2; 129, 129S6. *p < 0.05; ****p < 0.001.

Due to the lack of specificity of Dclk1 in identifying tuft cells, we evaluated expression of additional tuft cell markers Pou2f3 and Trpm5 (Figures 6A,B). Pou2f3 expression is absent from the normal pancreas, but has been reported to occur in response to activation of *Kras^*G*12*D*^* (Supplementary Figure S6; Zhang et al., 2018). Consistent with the literature, Trpm5 can be detected in the islets of the normal pancreas; however, expression is absent in the exocrine portion of the tissue (Supplementary Figure S6; Colsoul et al., 2010). In response to four cycles of caerulein, we observed a significant increase in Pou2f3 expression in 4 of the 7 strains analyzed. The Pou2f3+ strains also presented with Trpm5 expression consistent with tuft cell formation. While 97% of pancreatitis tuft cells are Pou2f3 positive (189/195 cells, *n* = 4 mice), only 81% of Pou2f3 positive cells (118/145 cells, *n* = 4 mice) were determined to be tuft cells by co-expression of marker Cox1 (conducted in the CD-1 strain). Consistent with this analysis, 79% of Pou2f3+ cells (239/302 cells, *n* = 4 mice) were positive for acetylated α-tubulin (which labels the dense tubulin network found in tuft cells). To confirm tuft cell formation, we conducted co-immunofluorescence for markers Dclk1, Cox1, and acetylated α-tubulin on treated and control pancreata from all seven strains. By this analysis we confirmed that the CD-1, Swiss Webster, DBA/2, and 129S6 strains do, in fact, form tuft cells in response to chronic injury (Figure 6C). These data demonstrate that mouse strains differ significantly in their susceptibility to tuft cell formation in response to injury.

### The Epithelial and Stromal Composition of Pancreatitis Varies by Mouse Strain

**FIGURE 7:**
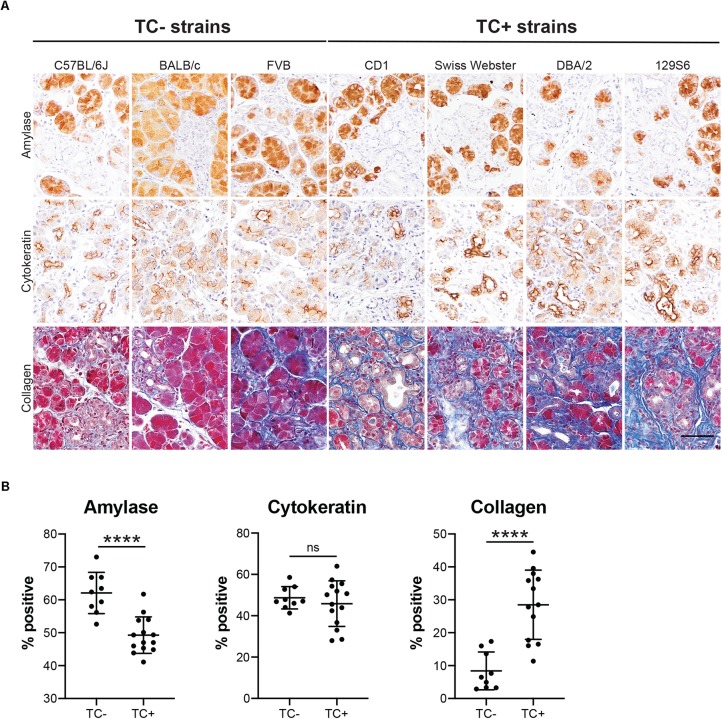
Epithelial and stromal marker expression in caerulein-induced pancreatitis varies by mouse strain. **(A)** IHC for acinar cell marker amylase or ductal cell marker cytokeratin (both brown), or collagen staining (Trichrome, blue) in the pancreata of mice treated with four cycles of caerulein, quantified in **(B)**. Scale bar, 50 μm. TC–, measurements from mouse strains lacking tuft cells (C57BL/6J, BALB/c, and FVB); TC+, measurements from mouse strains that form tuft cells in response to caerulein (CD1, Swiss Webster, DBA/2, 129S6). ****p < 0.01; ns, not significant.

**FIGURE 8:**
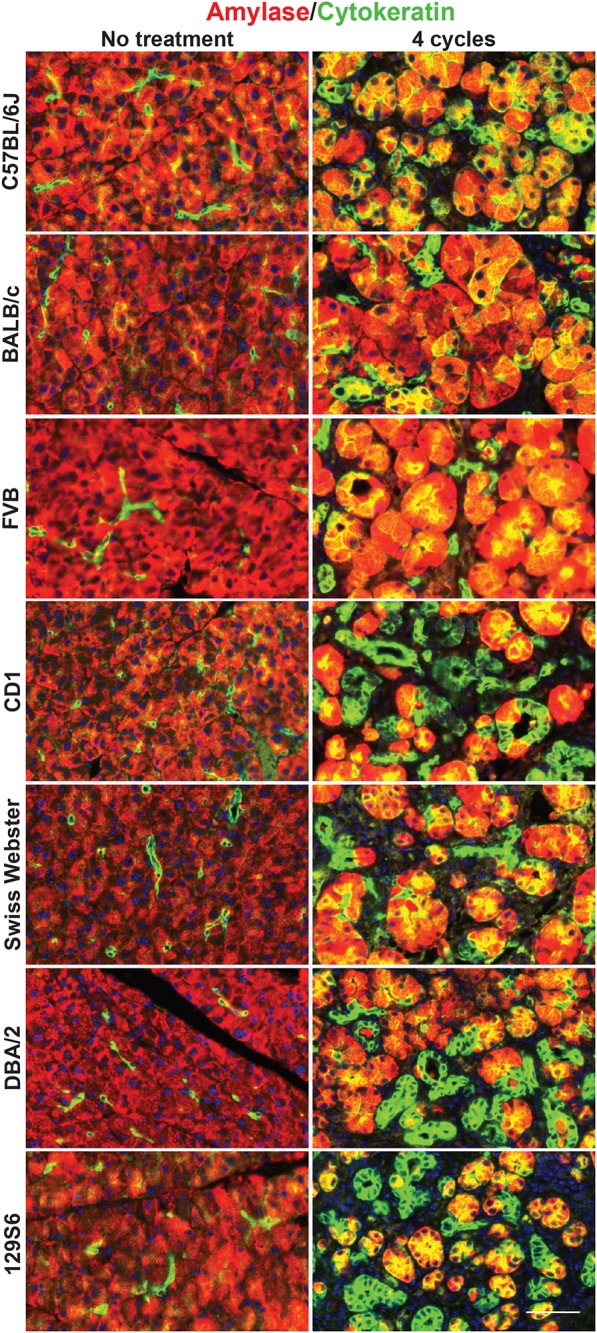
Co-expression of acinar and ductal markers in the normal or injured pancreata of wild type mice. Immunofluorescence for acinar cell marker amylase (red) or ductal cell marker cytokeratin (green) in the pancreata of untreated or treated (four cycles of caerulein) mice from all seven strains. Cells co-positive for amylase and cytokeratin appear yellow. Scale bar, 50 μm.

Our lineage tracing analyses, conducted in CD-1 mice, demonstrate that nearly all pancreatitis tuft cells result from ADM (Figure 4). These results led us to hypothesize that the proclivity for acinar cells to undergo this cell state reprograming varies between mouse strains. As an initial test of this hypothesis we compared the relative area occupied by acinar and ductal cells in caerulein-treated pancreata from all seven strains. Amylase is a digestive enzyme produced by acinar cells, while cytokeratin is a structural protein characteristic of ductal cells. Consistent with this hypothesis, we found significantly less amylase expression in mouse strains that form tuft cells in response to injury (48.7 ± 3.8%; CD1, Swiss Webster, DBA/2, 129S6; *n* = 5 for CD1, *n* = 3 for all other strains) vs. those strains that do not (62.1 ± 5.6%; C57BL/6J, BALB/c, FVB; *n* = 3 each) (Figures 7A,B and Supplementary Figure S7). Cytokeratin expression, however, was not significantly different between the two groups (47.88 ± 9.9% for tuft cell positive stains vs. 48.1 ± 3.4% for tuft cell negative strains) (Figures 7A,B and Supplementary Figure S7). Interestingly, we found that TC+ strains are characterized by significantly more stromal deposition (collagen, 28.49 ± 10.52%; *n* = 4 for CD1, *n* = 3 for all other strains) than TC− strains (8.39 ± 5.74%; *n* = 3 for all strains) (Figures 7A,B and Supplementary Figure S7). When we evaluated co-expression of amylase and cytokeratin, we were surprised to find cells with acinar cell morphology that co-expressed both markers in the caerulein-treated pancreata from all seven strains, regardless of tuft cell status (Figure 8). Collectively, these data indicate that acinar cells up-regulate cytokeratin expression in response to injury, but do not necessarily undergo metaplasia (ADM). While this is suggestive of varying proclivity for ADM between strains, lineage tracing in each strain will be required to evaluate this hypothesis more thoroughly.

## Discussion

Our data show that tuft cell formation occurs in genetically wild type mice as part of the epithelial response to chronic pancreatic injury, and that this process is highly strain dependent. While tubulin kinase Dclk1 is a fairly specific tuft cell marker in the intestines, our data clearly show that non-tuft cells express Dclk1 under conditions of pancreatic injury, consistent with previous studies (Westphalen et al., 2016). Consequently, multiple markers must be used to confirm tuft cell formation, and additional analysis by electron microscopy provides the gold standard for unambiguous identification.

Our studies reveal that nearly all pancreatitis tuft cells arise from acinar cells, identifying a new layer of epithelial cell plasticity in pancreatic injury. One implication of this discovery is that tuft cells play a role in pancreatitis progression and/or recovery from injury. Previous studies have shown that in response to injury and regeneration, existing acinar cell populations mainly drive the genesis of new exocrine tissue rather than ADM-derived ductal cells (Desai et al., 2007; Strobel et al., 2007). This begs the question of the function of these morphologically distinct and highly specialized cells in pancreatitis. Consistent with a function in recovery, we found that, while two cycles of caerulein treatment is insufficient to induce tuft cell formation, pancreatic tuft cells will form in these mice during the recovery phase (data not shown).

We also show that pancreatitis tuft cells express cytokine IL-25. In the gut, it has been shown that tuft cell-derived IL-25 is required to mount a Th2 immune response to parasite infection. Tuft cell-derived IL-25 stimulates group 2 innate lymphoid cells (ILC2s) to produce IL-5, -9, and -13, promoting type 2 inflammation (Ting and von Moltke, 2019). IL-13 is sufficient to skew the lineage of undifferentiated intestinal epithelial cells toward tuft and mucin-producing goblet cells. Goblet cells then secrete large amounts of mucus, which aids in clearance of the infection (Gerbe et al., 2016; Howitt et al., 2016; von Moltke et al., 2016). While the pancreas is devoid of goblet cells, pancreatitis is characterized by the formation of mucin-producing cells, which may play a role in neutralizing injury and promoting epithelial recovery. Further, IL-25 itself has been shown to suppress tumorigenesis in a number of cancer models, including PDA, consistent with a protective role for tuft cells in pancreas disease (Benatar et al., 2010). While, IL-25 is an attractive candidate to study tuft cell function in pancreatitis, our RNA-seq analysis identified a number of other potential candidates, which also play a role in mediating inflammation and deserve further investigation (Supplementary File S1).

Interestingly, we found significant differences between mouse strains in their ability to form tuft cells in response to the same stimuli. All seven strains analyzed experience pancreatitis in response to chronic caerulein treatment, as evidenced by a significant decrease in the pancreas to body weight ratio and inflammation identified histologically, but epithelial heterogeneity was substantially different. It is well reported in the literature that different mouse strains, and even different mouse sub-strains, react to caerulein-induced injury with disparate immune infiltrates and varying severity of disease (Wang et al., 2010; Ulmasov et al., 2013). The root of these differences lies in the intrinsic genetic and epigenetic variations between strains. This poses interesting implications for susceptibility to pancreatitis in patients. This report, to the best of our knowledge, is the first to demonstrate strain-dependent tuft cell formation and susceptibility to ADM. As tuft cells likely play an important role in pancreatitis, genetic susceptibility to tuft cell formation may represent a critical factor in pancreatitis formation, severity, and progression in patients.

## Data Availability Statement

The data generated in this study has been deposited in the Gene Expression Omnibus (Accession No. GSE143749).

## Ethics Statement

The animal study was reviewed and approved by Iacuc, the Salk Institute for Biological Studies.

## Author Contributions

KD and GW contributed to the conceptualization. KD, RN, LF, C-YC, and CO’C contributed to the formal analysis. KD, TH, UM, and GW contributed to the funding acquisition and supervision. KD, RN, CR, and NL contributed to the investigation. KD and RN contributed to the project administration. TH, UM, and GW contributed to the resources. C-YC, LF, and UM contributed to the software. KD, RN, and NL contributed to the visualization.

## Conflict of Interest

The authors declare that the research was conducted in the absence of any commercial or financial relationships that could be construed as a potential conflict of interest.
